# System-Level and Granger Network Analysis of Integrated Proteomic and Metabolomic Dynamics Identifies Key Points of Grape Berry Development at the Interface of Primary and Secondary Metabolism

**DOI:** 10.3389/fpls.2017.01066

**Published:** 2017-06-30

**Authors:** Lei Wang, Xiaoliang Sun, Jakob Weiszmann, Wolfram Weckwerth

**Affiliations:** ^1^Department of Ecogenomics and Systems Biology, University of ViennaVienna, Austria; ^2^Vienna Metabolomics Center, University of ViennaVienna, Austria

**Keywords:** *Vitis vinifera*, berry development, mass spectrometry, primary metabolism, secondary metabolism, flavonoids, systems biology, data integration

## Abstract

Grapevine is a fruit crop with worldwide economic importance. The grape berry undergoes complex biochemical changes from fruit set until ripening. This ripening process and production processes define the wine quality. Thus, a thorough understanding of berry ripening is crucial for the prediction of wine quality. For a systemic analysis of grape berry development we applied mass spectrometry based platforms to analyse the metabolome and proteome of Early Campbell at 12 stages covering major developmental phases. Primary metabolites involved in central carbon metabolism, such as sugars, organic acids and amino acids together with various bioactive secondary metabolites like flavonols, flavan-3-ols and anthocyanins were annotated and quantified. At the same time, the proteomic analysis revealed the protein dynamics of the developing grape berries. Multivariate statistical analysis of the integrated metabolomic and proteomic dataset revealed the growth trajectory and corresponding metabolites and proteins contributing most to the specific developmental process. K-means clustering analysis revealed 12 highly specific clusters of co-regulated metabolites and proteins. Granger causality network analysis allowed for the identification of time-shift correlations between metabolite-metabolite, protein- protein and protein-metabolite pairs which is especially interesting for the understanding of developmental processes. The integration of metabolite and protein dynamics with their corresponding biochemical pathways revealed an energy-linked metabolism before veraison with high abundances of amino acids and accumulation of organic acids, followed by protein and secondary metabolite synthesis. Anthocyanins were strongly accumulated after veraison whereas other flavonoids were in higher abundance at early developmental stages and decreased during the grape berry developmental processes. A comparison of the anthocyanin profile of Early Campbell to other cultivars revealed similarities to Concord grape and indicates the strong effect of genetic background on metabolic partitioning in primary and secondary metabolism.

## Introduction

Grapevine (*Vitis vinifera* L.) is one of the most important and widely cultivated economic crops. Grape berries are consumed either as fresh fruit or processed to raisins, juice and wine. Besides its enormous economical and nutritional values, grapes and grape products possess a wide variety of health benefits, such as antioxidation (Sánchez-Moreno et al., 1999; Doshi et al., 2006; Sánchez-Alonso et al., 2007; Sáyago-Ayerdi et al., 2009; Anastasiadi et al., 2010), cardiovascular protection (Tebib et al., 1994; Adisakwattana et al., 2010; Razavi et al., 2013), neuroprotection (Feng et al., 2007), anti-obesity (Kim et al., 2013; Zhang et al., 2013), etc.

The grape berry is a non-climacteric fruit. From fruit set to ripening, grape berries undergo three main developmental phases including two sigmoidal growth phases with an intermediate lag phase (Kennedy, 2002). The performance of grape berry development is characterized by dramatic changes in both physiology and biochemistry, including increases in volume and weight, changes in texture, color, aroma, acidity, sugar contents, susceptibility to disease, etc. The first growth phase (phase I) is characterized by fruit formation and enlargement due to the active cell division and expansion. In this phase, a notable accumulation of organic acids, especially malic and tartaric acid has been observed (Conde et al., 2007). Phase II, which is defined as a lag phase features a slow enlargement of berry volume caused by a stop in cell division. The grape berry is still green and hard at this phase. Organic acids continuously accumulate until veraison, which marks the beginning of phase III. During the last phase, the grape berries undergo a second sigmoidal growth accompanied by a decrease in acidity and increase in sugar content (Conde et al., 2007; Deluc et al., 2007; Fortes et al., 2011; Liang et al., 2011; Dai et al., 2013; Degu et al., 2014; Fraige et al., 2015; Cuadros-Inostroza et al., 2016). The peel of red varieties colors as a result of the accumulation of anthocyanins (Boss et al., 1996; Ali M. B. et al., 2011; Degu et al., 2014; Fraige et al., 2015). The grape berry becomes soft in the final phase and is ready to be harvested. Another generally adopted descriptive system is the E-L system which was proposed firstly by Eichhorn and Lorenz (1978) with a more detailed description of grape berry development stages.

Fruit development is an intricate process, featuring complex regulation and fine-tuned changes in metabolism. Its analysis requires the use of sensitive methods, which allow high sample throughput to cope with the amount of samples necessary to examine a time continuous process.

Since the release of the grapevine genome sequence in 2007 (Jaillon et al., 2007; Velasco et al., 2007), studies of developing grape berry based on transcriptomic (Deluc et al., 2007; Palumbo et al., 2014), proteomic (Giribaldi et al., 2007; Negri et al., 2008; Martinez-Esteso et al., 2011; Fraige et al., 2015) and metabolomic (Ali K. et al., 2011; Dai et al., 2013; Degu et al., 2015) techniques contributed extensively to our understanding of berry growing and ripening process. These studies not only enhanced and supplemented the morphological and physiological descriptions but also promoted the work to molecular level. Exploring the developmental process basing on a single level data results in a partial view of the progress. Several studies described the developmental process by combining transcriptomic and metabolomic profiles (Fortes et al., 2011; Agudelo-Romero et al., 2013; Degu et al., 2014). Considering that the proteome is the active part of the metabolic phenotype, integration and complex statistical correlation network analysis of those data will provide crucial information for the understanding of the metabolic and physiological changes (Weckwerth et al., 2004b; Morgenthal et al., 2005; Wienkoop et al., 2008; Valledor et al., 2013, 2014; Nukarinen et al., 2016; Wang et al., 2016a,c). Nonetheless, systematic analysis of integrated metabolome and proteome profiles of developing grape berries is still less covered. It is also problematic to schematize the metabolic dynamics of developing grape berry by summarizing or comparing those studies due to the coverage limitation of either developmental stages or metabolism branches. For instance, some studies only focus on primary metabolism (Dai et al., 2013) whereas others target flavonoid accumulation during grape berry ripening (Ali M. B. et al., 2011). Zamboni and coworkers integrated the transcriptomic, proteomic and secondary metabolite data of four developmental and three postharvest time points of Corvina grape berry into a complex statistical correlation network analysis for the identification of putative, stage-specific biomarkers (Zamboni et al., 2010). In addition, some studies worked on individual parts of grape berries, such as skin (Negri et al., 2008; Ali M. B. et al., 2011; Degu et al., 2014, 2015; Wu et al., 2014) or berries depleted of seed or peel (Martinez-Esteso et al., 2011; Fang et al., 2013; Fraige et al., 2015).

In this study, we harvested samples according to the modified E-L system (Coombe, 1995) from fruit set to ripening at 12 time points. Mass spectrometry based high-throughput platforms were applied for the metabolomic and proteomic analysis of both primary and secondary metabolism dynamics of developing grape berries. Multivariate statistical analysis of the dynamics of metabolites and proteins involved in primary metabolism i.e., glycolysis, tricarboxylic acid (TCA) cycle, amino acid metabolism as well as secondary metabolism i.e., flavonol, flavan-3-ols, anthocyanins and lignin unveiled metabolism interactions during the berry growing period.

## Materials and methods

### Sample collection

**Figure 1:**
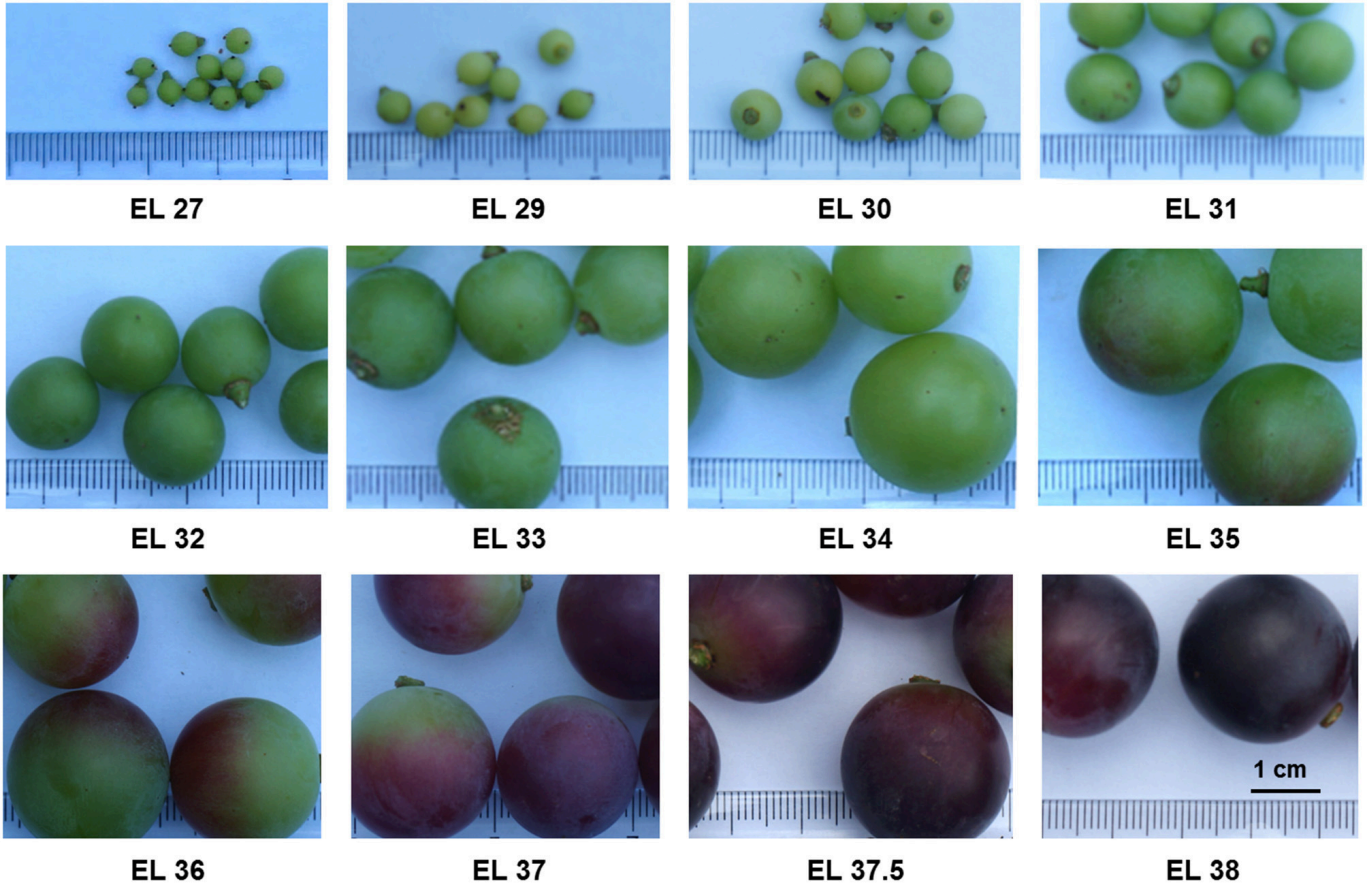
Grape berries harvested at 12 developmental stages according to the modified E-L system.

Berries at 12 developmental stages corresponding to EL 27, 29, 30, 31, 32, 33, 34, 35, 36, 37, 37.5 (to distinguish with the samples at early EL 37 stage), 38 (Figure 1) were harvested according to the modified Eichhorn-Lorenz system (E-L system) (Eichhorn and Lorenz, 1978; Coombe, 1995) from *V. vinifera* (Early Campbell) growing in the plant garden of University of Vienna (48°13′50.2″N 16°21′28.2″E) during the 2014 growing year. The plants did not receive any specific training system. Three biological replicates each containing 5 to 10 berries were collected for each developmental phase. The harvested berries were frozen in liquid nitrogen immediately and stored at −80°C.

### Metabolite and protein extraction

An integrative extraction of metabolites and proteins was performed according to a universal extraction protocol (Weckwerth et al., 2004b) with some modifications. The grape berries were ground to fine powder in liquid nitrogen using mortar and pestle. 50 to 100 mg of material was extracted with 750 μl of extraction solution (methanol: water: formic acid = 70:28:2) and 250 μl of hexane. The mixture was homogenized by vigorous vortexing and incubated 30 min on ice. Then the mixture was centrifugated at 20,000 g for 8 min to separate the lipophilic and hydrophilic phases which were subsequently transferred into new tubes, respectively. The extraction procedure was repeated once with the lipophilic and hydrophilic phases pooled together with those from the first extraction, respectively. The extracts were dried under vacuum. Proteins were extracted from the residue pellets according to a previous protocol (Noah et al., 2013).

### Metabolite measurement, identification and quantification

The dried hydrophilic phases were re-dissolved in 400 μl of 50% methanol. For the primary metabolite analysis, 25 μl of this re-dissolved hydrophilic phase was dried under vacuum and subsequently derivatized according to a modified protocol (Weckwerth et al., 2004b; Mari et al., 2013). Agilent® 6890 gas chromatograph coupled to a LECO Pegasus® 4D GC × GC-TOF spectrometer was used for the primary metabolite measurement. Instrument parameters were set as described previously (Doerfler et al., 2013). GC separation was performed at a constant flow 1 mL min^−1^ helium. Initial oven temperature was set to 70°C and hold for 1 min, followed by a ramp to 76°C at 1°C min^−1^ and a second ramp at 6°C min^−1^ to 350°C hold for 1 min. Transfer line temperature was set to 340°C and post run temperature to 325°C for 10 min. The metabolite identification and quantification was performed with LECO Chroma TOF®. Retention times (RTs) of the peaks were converted to retention indices (RIs) according to the RTs of spiked alkanes (C12-C40). Metabolites were annotated by comparing their RIs and mass spectra to those of standards in the GMD Golm database (Kopka et al., 2005) with a minimum match factor set to 700. The peak areas of the annotated metabolites corresponding to specific masses were extracted and used for relative quantification. Mixtures of standard compounds were measured under the same conditions at different concentrations to calculate the standard curves for absolute quantification.

For the secondary metabolite analysis, 10 μl of the re-dissolved hydrophilic phase was mixed with 2.5 μl of reserpine (5 mg l^−1^) as an internal standard, 10 μl of 1.0% formic acid (FA) solution and 77.5 μl of water. After centrifugation at 20,000 g for 8 min, 5 μl of the supernatant were loaded on Waters ACQUITY UPLC HSS T3 nanoACQUITY Column (particle size 1.8 μm, dimension 100 μm × 100 mm) via a HTC PAL Autosampler device coupled to an Eksigent nano LC pump and eluted with a non-linear gradient (Mari et al., 2013) at a constant flow rate of 500 nl min^−1^. The LC conditions were 5% B during 0–3 min, a linear increase from 5 to 20% B during 3–25 min, from 20 to 40% B during 25–40 min and from 40 to 50% B during 40–55 min, finally from 50 to 95% B during 55–63 min followed by 15 min of maintenance with a flow rate of 500 nl min^−1^. Ionization was performed by a nano ESI source (Thermo Scientific, USA) in positive mode with the masses analyzed by a LTQ Orbitrap XL™ mass spectrometer (Thermo, Germany). Each full scan was followed by one MS/MS scan with the most abundant precursor ion fragmented by collision induced dissociation (CID) under 35% of the normalized collision energy during 90 ms activation time. The minimum signal threshold was set to 50,000. Before measurement, the machine was calibrated and standards were measured to check the condition of measurement. We also ensured linearity of the spiked internal standard in different concentrations. The combination of a very low flow rate (500 nL/min) and a gradient that minimized co-elution was chosen to minimize matrix effects. For secondary metabolite identification, accurate precursor masses, sum formula RTs together with mass accuracy were exported from Xcalibur (Thermo Xcalibur 2.2 SP1.48) and compared with the information from literature or standard compounds. The annotation levels were marked according to a standard proposed by the Metabolomics Standards Initiative (Sumner et al., 2007). LCquan (Thermo, v2.6.6.1128) was used for peak extraction and peak area integration.

### Protein digestion and analysis

Protein concentration was determined by the Bradford method (Bradford, 1976) with a BSA standard curve. 100 μg of protein were firstly reduced with dithiothreitol (DTT, 5 mM, 37°C, 45 min); then alkylated with iodoacetamide (IAA, 10 mM, 23°C, dark, 60 min) and finally 5 mM of DTT was added (23°C, dark, 15 min). Endoproteinase LysC and trypsin were applied for digestion based on a previous protocol (Hoehenwarter et al., 2008). After digestion, samples were desalted with C18-SPEC-96 well plate (15 mg, Agilent, USA) according to the manufacturer's instruction. The eluted peptides were dried under vacuum and dissolved in 500 μl of start gradient solution (4.5% acetonitrile, 0.1% FA). 1 μg of the digested protein was loaded on an Ascentis Peptide ES-C18 column (particle size 2.7 μm, dimension 15 cm × 100 μm, Sigma-Aldrich, USA) and eluted with a 90 min linear gradient from 5 to 40% of mobile phase B (90% acetonitrile, 0.1% FA; phase A, 0.1% FA in water) at a constant flow rate of 400 nl min^−1^. The same ESI-LTQ-Obitrap equipment used for metabolite analysis was applied for peptide measurement. Each full scan was followed by 10 MS/MS scans in which the 10 most abundant ions were selected and fragmented by CID with 35% of the normalized collision energy during a 30 ms activation time. Minimum signal threshold was set to 10,000.

The obtained raw files containing peptide information were searched against a grape fasta file including 65,448 protein sequences from UniProt with the SEQUEST algorithm in Proteome Discoverer (v 1.3, Thermo Scientific). Searching parameters were set as below: maximum two missed cleavage sites, acetylation for N-terminal modification, oxidation of methionine for dynamic modification and carbamidomethylation of cysteine for static modification were allowed. Mass tolerance for precursors was set to 10 ppm and for fragment masses to 0.8 Da. False discovery rate (FDR) was set to 0.01. Protein candidates were defined by at least two peptides with high confidence. The obtained raw files and sequence information of the identified proteins were submitted to the public repository ProteomeXchange (Vizcaino et al., 2014) with the dataset identifier PXD003769 (http://www.proteomexchange.org/) as well as to the PROMEX database (http://promex.pph.univie.ac.at/promex/). Normalized spectral abundance factors (NSAFs) were calculated (Zybailov et al., 2006) for relative quantification. The protein candidates that are present in all the three biological replicates of at least one stage were considered for the statistical analysis. For the functional analysis, the identified protein sequences were blasted against a protein database of *Arabidopsis thaliana* (from PLAZA with 27,407 protein sequences) and *Theobroma cacao* (from PLAZA with 44,404 protein sequences) with the BLASTP function in NCBI (v 2.2.31, ftp://ftp.ncbi.nlm.nih.gov/blast/executables/blast+/LATEST/). A cacao mapping file from GoMapMan (“tca_Phytozome9.1_transcript_2015-01-09_mapping.xlsx,” http://www.gomapman.org/export/current/mapman) was applied for the functional analysis.

### Statistical analysis

The obtained metabolite data were normalized to fresh weight and dodecane (C12 alkane, GC-MS data) or total ion intensity (LC-MS data). Analysis of variance (ANOVA) and k-means clustering were performed within Matlab® (V8.4.0 R2014b; http://www.mathworks.com). The significant levels of the candidates were presented with lower case letters according to results of the Duncan's test (Duncan, 1955). K-means clustering analysis was repeated 100 times and finally the result with minimal total distance was selected. Principal component analysis (PCA), hierarchical clustering analysis and Granger causality analysis were performed with COVAIN under Matlab environment (Sun and Weckwerth, 2012). Granger causality analysis was performed on all the identified metabolites and proteins as well as the clusters after k-means clustering analysis with their time lag was set to 1, 2, 3, respectively. The correlations with *p*-values less than 0.05 were recorded. The network of Granger result was visualized in Cytoscape (http://www.cytoscape.org/). The Venn diagram was drawn with Venny 2.0.2 (http://bioinfogp.cnb.csic.es/tools/venny/).

## Results

### Metabolomic profiles of developing grape berry

**Figure 2:**
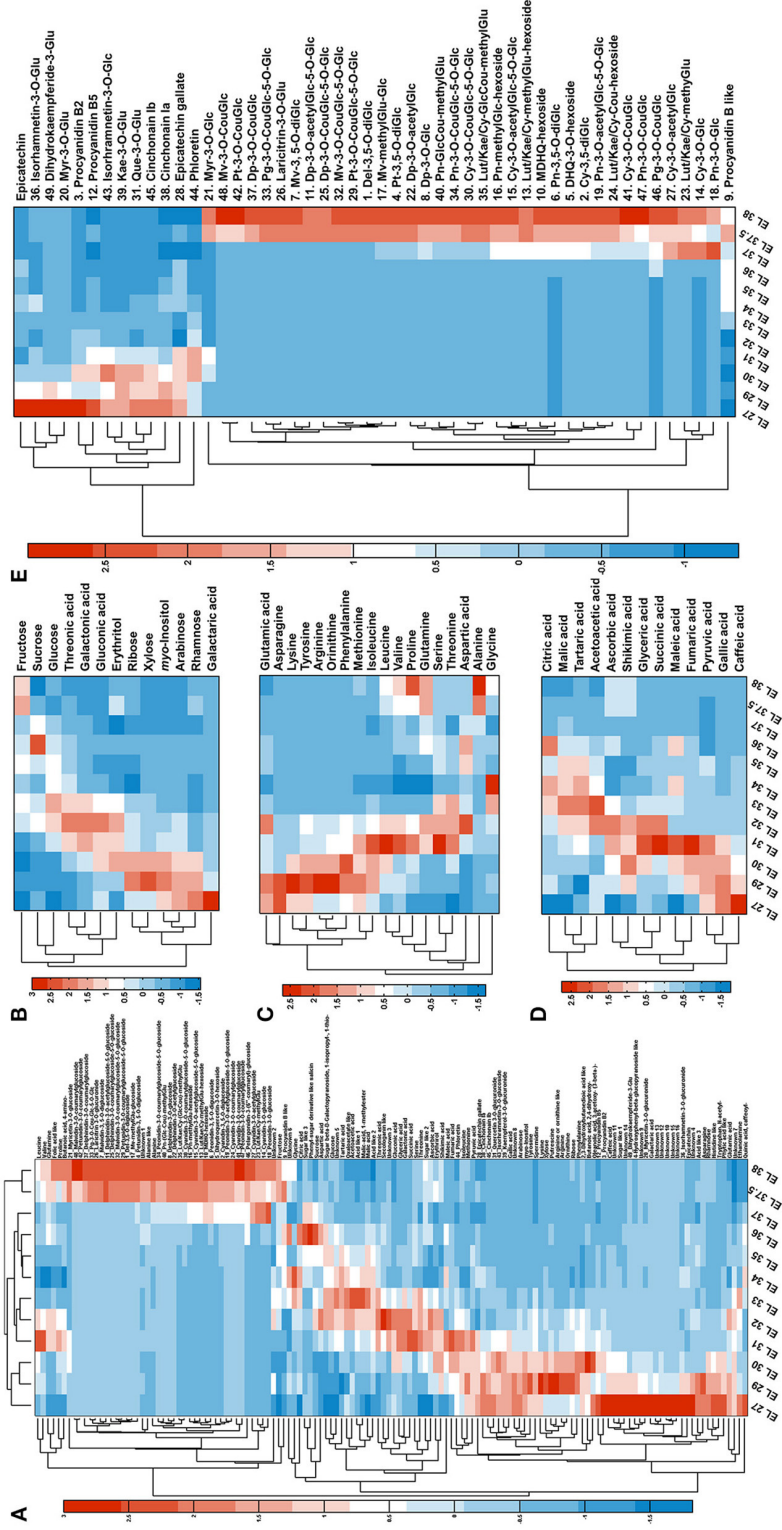
Metabolome dynamics of developing grape berry. **(A)** Overview of the metabolite dynamics with bi-hierarchical-clustering heat map. **(B–D)** Present the dynamics of sugars (including sugar alcohol and sugar acids), amino acids and organic acids. **(E)** Presents the dynamics of flavonoids.

The GC-TOF-MS platform allowed the annotation of 87 candidates including sugars, amino acids, organic acids together with simple amine and phenolic compounds, according to their RIs and mass spectra. Additionally, 49 flavonoids were annotated from LC-Orbitrap-MS data according to the accurate precursor masses, sum formula and their fragmentation patterns (Table 1). The detailed information (RI and RTs, quantification masses or MS2 fragments and the integrated peak area, one way ANOVA results) of the annotated candidates is listed in Table S1. The dynamic patterns of the annotated metabolites were visualized by a hierarchical bi-clustering color map (Figure 2A). Sugars (including sugar alcohols, sugar acids), amino acids, organic acids and flavonoids were further shown in Figures 2B–E.

**Table 1:** Flavonoids annotated from LC-MS data of developing grape berries.

**Candidate number**	**Deduced structure**	**RT (min)**	**[M+H]^+^ (m/z)**	**Mass accuracy (ppm)**	**Export formula**	**MS/MS (m/z)**	**References**	**Annotation level**
1	Dp-3, 5-O-diGlc	9.9	627.15536	−0.21	C27H31O17	465, 303	Liang et al., 2008; He et al., 2010	2
2	Cy-3, 5-O-diGlc	11.5	611.16031	−0.4	C27H31O16	449, 287	Liang et al., 2008; He et al., 2010	2
3	Procyanidin B2	12.07	579.14927	−0.43	C30H27O12	427, 409, 291, 247	Monagas et al., 2005	1
4	Pt-3, 5-O-diGlc	12.5	641.17117	−0.06	C28H33O17	479, 317	Liang et al., 2008; He et al., 2010	2
5	DHQ-3-O-hexoside	12.5	467.11823	−0.17	C21H23O12	449, 305	Flamini et al., 2015	2
6	Pn-3, 5-O-diGlc	14.25	625.17598	−0.33	C28H33O16	463, 301	Liang et al., 2008; He et al., 2010	2
7	Mv-3, 5-O-diGlc	14.7	655.18679	−0.09	C29H35O17	493, 331	Liang et al., 2008; He et al., 2010	2
8	Dp-3-O-Glc	14.9	465.10259	−0.16	C21H21O12	303	Liang et al., 2008	2
9	Procyanidin B like	15.58	579.14934	−0.37	C30H27O12	427, 289, 291, 409	Monagas et al., 2005	3
10	MDHQ-hexoside	15.91	481.13399	−0.06	C22H25O12	463, 319, 301	Abu-Reidah et al., 2015	3
11	Dp-3-O-acetylGlc-5-O-Glc	16.3	669.16632	0.18	C29H33O18	507, 303, 465	He et al., 2010	2
12	Procyanidin B5	16.61	579.1494	−0.31	C30H27O12	427, 409, 291, 247	Monagas et al., 2005	2
13	Lut/Kae/Cy-methylGlu-hexoside	16.7	639.15567	0.09	C28H31O17	477, 287, 449		3
14	Cy-3-O-Glc	16.88	449.10768	−0.16	C21H21O11	287	Liang et al., 2008	2
15	Cy-3-O-acetylGlc-5-O-Glc	17.73	653.17121	−0.02	C29H33O17	491, 287, 449	Liang et al., 2008; He et al., 2010	2
16	Pn-methylGlu-hexoside	18.46	653.17117	−0.06	C29H33O17	491, 301, 463		3
17	Mv-methylGlu-glucoside	18.46	683.18171	−0.08	C30H35O18	521, 331, 317, 493		3
18	Pn-3-O-Glc	19.65	463.12328	−0.21	C22H23O11	301	Liang et al., 2008	2
19	Pn-3-O-acetylGlc-5-O-Glc	20.32	667.18708	0.2	C30H35O17	505, 301, 463	He et al., 2012	2
20	Myr-3-O-Glu	22.69	495.07682	−0.11	C21H19O14	319	Flamini et al., 2015	2
21	Myr-3-O-Glc	22.85	481.09749	−0.18	C21H21O13	319	Flamini et al., 2015	2
22	Dp-3-O-acetylGlc	23	507.11316	−0.16	C23H23O13	303	Liang et al., 2008	2
23	Lut/Kae/Cy-methylGlu	23.21	477.10265	−0.21	C22H21O12	287		3
24	Lut/Kae/Cy-Cou-hexoside or Lut/Kae/Cy-Caff-Rha	23.74	595.14454	−0.07	C30H27O13	287	Liang et al., 2008; He et al., 2010	3
25	Dp-3-O-CouGlc-5-O-Glc	24.13	773.1922	−0.16	C36H37O19	611, 303, 465	Liang et al., 2008; He et al., 2010	2
26	Lar-3-O-Glu	24.8	509.09238	−0.2	C22H21O14	319	Flamini et al., 2015	2
27	Cy-3-O-acetylGlc	25.04	491.11828	−0.12	C23H23O12	287	Liang et al., 2008	2
28	Epicatechin gallate	25.17	443.09711	−0.16	C22H19O10	273, 151, 291	Delcambre and Saucier, 2012	2
29	Pt-3-O-CouGlc-5-O-Glc	25.76	787.20786	−0.15	C37H39O19	625, 317, 479	He et al., 2010	2
30	Cy-3-O-CouGlc-5-O-Glc	26.1	757.19691	−0.54	C36H37O18	595, 287, 449	Liang et al., 2008; He et al., 2010	2
31	Que-3-O-Glu	26.4	479.08192	−0.1	C21H19O13	303	Flamini et al., 2015	2
32	Mv-3-O-CouGlc-5-O-Glc	27.84	801.22316	−0.49	C38H41O19	639, 611, 493, 331, 303	Liang et al., 2008; He et al., 2010	2
33	Pg-3-O-Cou-Glc-5-O-Glc	28.11	741.20284	0.31	C36H37O17	579, 271, 433	He et al., 2012	2
34	Pn-3-O-CouGlc-5-O-Glc	28.37	771.21278	−0.31	C37H39O18	609, 301, 463	Liang et al., 2008; He et al., 2010	2
35	Lut/Kae/Cy-(GlcCou)-methylGlu	28.76	785.19222	−0.14	C37H37O19	287, 477, 595, 623		3
36	Isorhamnetin-3-O-Glu	28.96	493.09758	−0.08	C22H21O13	303	Flamini et al., 2015	2
37	Dp-3-O-CouGlc	29.27	611.13932	−0.21	C30H27O14	303	He et al., 2010	2
38	Cinchonain Ia	29.32	453.11789	−0.24	C24H21O9	343, 313, 301	Chen et al., 2015	2
39	Kae-3-O-Glu	29.42	463.08706	−0.04	C21H19O12	287	Flamini et al., 2015	2
40	Pn-(Glc-Cou)-methylGlu	30.29	799.20804	0.04	C38H39O19	609, 301, 491, 637		3
41	Cy-3-O-CouGlc	30.67	595.14423	−0.38	C30H27O13	287	Liang et al., 2008; He et al., 2010	2
42	Pt-3-O-CouGlc	30.98	625.15504	−0.14	C31H29O14	317	Liang et al., 2008; He et al., 2010	2
43	Isorhramnetin-3-O-Glc	31.2	479.11835	−0.05	C22H23O12	317	Flamini et al., 2015	2
44	Phloretin	31.38	275.09134	−0.23	C15H15O5	107, 169, 149, 127	Zhao et al., 2014	2
45	Cinchonain Ib	31.52	453.11784	−0.38	C24H21O9	343, 301, 313	Chen et al., 2015	2
46	Pg-3-O-Cou-Glc	31.91	579.1496	−0.1	C30H27O12	271	Castillo-Munoz et al., 2009	2
47	Pn-3-O-CouGlc	32.23	609.16016	−0.11	C31H29O13	301	Liang et al., 2008; He et al., 2010	2
48	Mv-3-O-CouGlc	32.23	639.17083	−0.01	C32H31O14	331	Liang et al., 2008; He et al., 2010	2
49	Dihydrokaempferide-3-Glu	35.83	479.11835	−0.06	C22H23O12	289		3

*Api, Apigenin; Caff, Caffeoyl; Cou, Coumaryl; Cy, Cyanidin; DHQ, Dihydroquercetin (taxifolin); Dp, Delphinidin; Glc, Glucose; Glu, Glucuronide; Kae, Kaempferol; Lar, Laricitrin; Lut, Luteolin; MDHQ, methyldihydroquercetin; Mv, Malvidin; Myr, Myricetin; Pg, Pelargonidin; Pn, Peonidin; Pt, Petunidin; Que, Quercetin; Rha, Rhamnoside; Tri, Tricetin*.

#### Sugars

The main sugars in grape berries are fructose, glucose and sucrose. In the present study, fructose constantly accumulated during development with significant increases before veraison and during ripening (Figure 2B). Glucose also significantly accumulated around veraison but declined afterwards (Figure 2B). The content of sucrose fluctuated during grape development (Figure 2B) with four inflection points at EL 30, 32, 34, and 36 respectively. Other sugars, sugar alcohols and sugar acids either decreased during development (ribose, xylose, myo-inositol, arabinose, rhamnose, galactaric acid) or showed the highest level at EL 32 (threonic acid, galactonic acid, gluconic acid, erythrirol) (Figure 2B).

#### Amino acids

Amino acids showed distinct dynamics during grape berry development (Figure 2C). Arginine and asparagine were the most abundant amino acids in young berries, alanine and glutamine in mature berries (Table S2). Lysine, tyrosine, arginine, ornithine and phenylalanine increased significantly from EL 27 to EL 29, however, decreased dramatically until the end of lag phase (EL 34) and remained at a relatively low level during the second sigmoidal growth period (Figure 2C). Asparagine was in high abundance at the first two developing stages followed by a dramatic decline from EL 29 to EL 30 then stayed in low level until the end. Other amino acids fluctuated during grape berry developing and all showed a turning point at EL 34 which is the end of the lag phase and the beginning of the veraison (Figure 2C).

#### Organic acids

The predominant organic acids detected in grape berry were malic acid, tartaric acid and citric acid which increased before veraison (EL 35) and decreased afterwards (Figure 2D). Other organic acids showed similar changing pattern except pyruvic acid, gallic acid and caffeic acid which were highest in the young berries and then decreased throughout the developmental process (Figure 2D).

#### Flavonoids

Grape and its products are rich in polyphenolics. These secondary metabolites, especially flavonoids, play multiple roles in grape and attract more and more attentions due to their health benefits (Anastasiadi et al., 2010; Kim et al., 2013; Zhang et al., 2013). During grape berry development, the detected flavonoids presented two distinct changing patterns (Figure 2E). All the anthocyanins accumulated during ripening whereas most of the candidates in the other subfamilies like proanthocyanins, flavan-3-ol, flavonol, flavanonol and their glycosides were abundant in young berries and decreased during the time course of development (Figure 2E). The synthesis of anthocyanins splits into three branches, i.e., the monohydroxylated (pelargonidin, Pg), the dihydroxylated (cyanidin, Cy), and the trihydroxylated (delphinidin, Dp) branch. Cy and peonidin (Pn) glycosides which belong to the dihydroxylated branch were detected from stage EL 37 or even EL 36 whereas derivatives of the other two branches started to appear one stage later. Furthermore, in mature berries (EL 38), the relative abundance of Cy- and Dp- derivatives were higher than the corresponding derivatives of other aglycones (Figure S1). For instance, Cy-Cou-diGlc (**30**) and Dp-Cou-diGlc (**25**) were more abundant than petunidin- (Pt, **29**), malvidin- (Mv, **32**) and Pg- (**33**) coumaroyl-diglucoside; Cy-Glc (**2**) and Dp-Glc (**8**) were in higher level than glucoside of other aglycones (Figure S1).

### Protein profiles of developing grape berry

In total, 1313 proteins were identified from all the samples (for sequences information see in Table S3). 848 candidates prevalent in all replicates of at least one stage were used for further statistical analysis. NSAFs and the ANOVA analysis result can be found in Table S4. The functions of all the protein candidates were annotated by blasting against protein sequences of *A. thaliana* and *T. cacao*. Blast results were summarized in Table S4. The matching with the *T. cacao* database yielded a higher amount of hits with a better blast quality and was therefore used for functional analysis. Subsequently, the identified protein candidates were assigned to corresponding functional bins according to the cacao mapping file from GoMapMan (Table S4).

**Figure 3:**
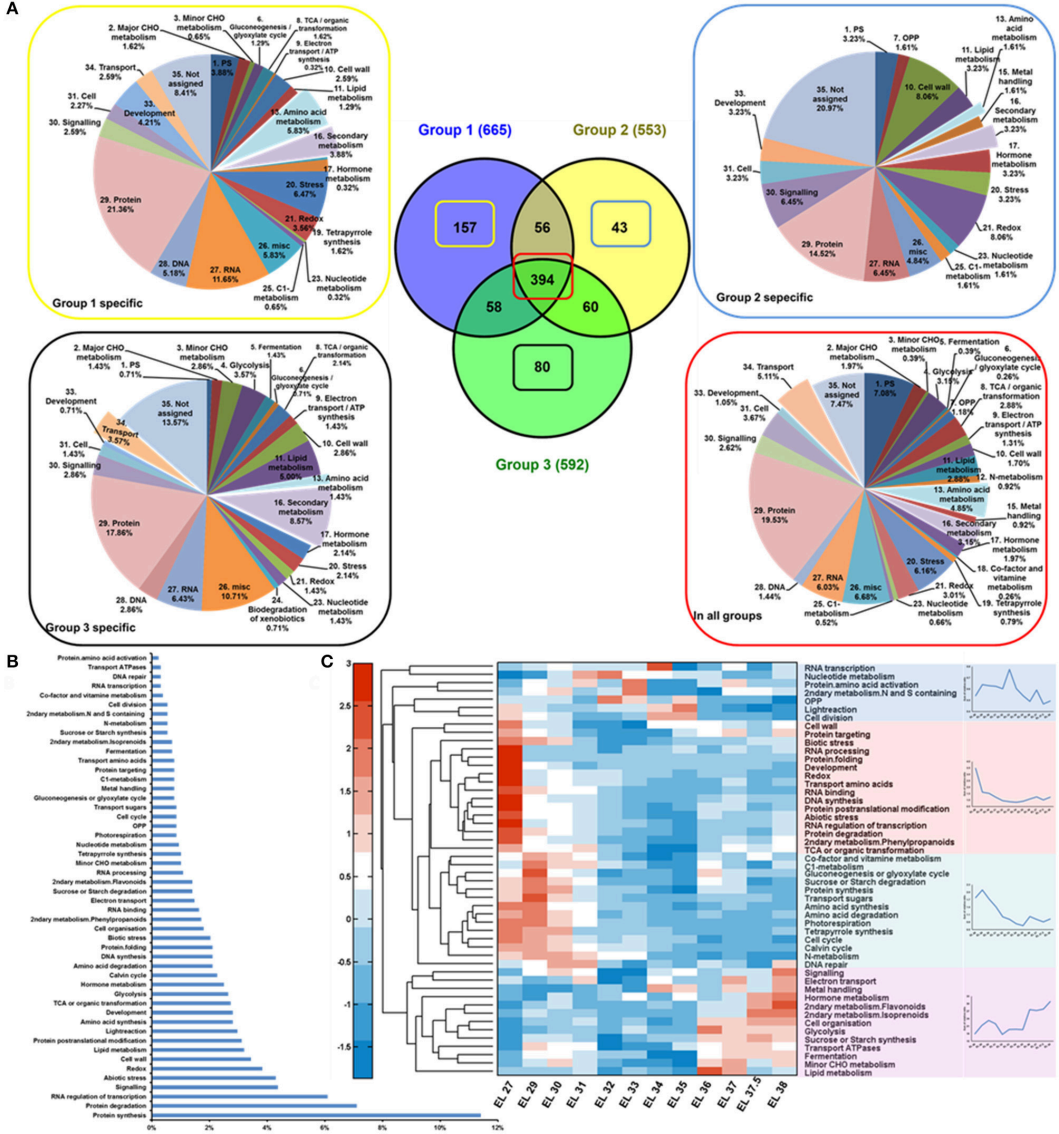
Proteomic analysis of developing grape berries. **(A)** Protein distribution throughout the developmental process. Samples of 12 developing stages were sorted to 3 groups with group 1 including samples at stage EL 27, 29, 30, 31; group 2, EL32, 33, 34, 35 and group 3, EL 36, 37, 37.5, 38. The proteins that specific to group 1, 2, 3 and those common to all groups were functionally summarized with pie charts in yellow, blue, black and red box respectively. **(B)** Protein frequencies. **(C)** Protein dynamics. Four distinctive changing patterns were summarized with line charts.

Hierarchical bi-clustering analysis was applied to visualize the dynamic proteome profiles of developing grape berry (Figure S2). Samples of 12 developing stages were clustered into 3 groups indicated with color blue, red and green (Figure S2). Samples from stage EL 27, 29, 30 and 31 were assigned to group 1; EL 32, 33, 34, 35 group 2 and EL 36, 37, 37.5, 38 group 3. The Venn diagram (Figure 3A) shows 394 proteins were common to all groups and 157, 43 and 80 protein candidates are specific to group 1, 2, and 3, respectively. The functional distribution of these group specific proteins was summarized with pie charts (Figure 3A). There were 18 amino acid metabolism related proteins detected accounting for 5.83% of all the group 1 specific proteins whereas only 1 and 2 proteins were accounting for 1.61 and 1.43% respectively of group 2 and 3 (Figure 3A) indicating active amino acid metabolism at phase I on proteome level. There were 8 transport related proteins accounting for 2.59% of group 1 specific proteins and 5 accounting 3.57% of group 3 specific proteins whereas there was no transport related protein exclusive to group 2 (Figure 3A). Another notable point is that more proteins associated with secondary metabolism existed exclusively in group 1 (12, 3.88%) and 3 (12, 8.57%) than in group 2 (2, 3.23%) (Figure 3A) indicating the synthesis of secondary metabolites was more active in the beginning and the end of berry developmental stages than in the middle.

The annotated protein candidates were assigned to 50 functional bins (Table S4). The majority of functional categories include candidates involved in protein synthesis (11.41%), protein degradation (7.11%), RNA regulation of transcription (6.09%), signaling (4.38%), and abiotic stress response (4.30%) (Figure 3B). The changing patterns of the proteins in these functional categories were summarized into four groups by hierarchical clustering analysis (Figure 3C) with their summarized changing patterns shown on the right side. Proteins in 30 out of 50 functional bins were in lowest content around veraison (Figure 3C in red and green). In contrast, proteins involved in 7 functional groups were with highest abundance just before veraison (EL 33) (Figure 3C in blue). The functional bins in purple group (Figure 3C) involve proteins constantly accumulating during grape berry development.

Proteins related to abiotic and biotic stresses (4.30 and 2.03%, respectively) showed high abundance at early developmental stages or/and during ripening (Table S3, Figure 3C in red) indicating high resistance ability of grape berry against environmental and developmental stresses during these developmental phases. A larger amount of oxidative stress responsive proteins in young, green berries as well as increasing expression of pathogen responsive proteins after veraison have been previously reported (Giribaldi et al., 2007). Additionally, another study reported a parallel transcript profile of stress/pathogens responsive gene strongly expressed in ripening berries (Davies and Robinson, 2000).

Photosynthesis (6.1%) is another major functional category including candidates involved in light reaction (2.97%), photorespiration (0.86%), and calvin cycle (2.27%) (Figure 3B). Proteins involved in light reaction were in higher abundance in the earliest stage and around veraison (Figure 3C in blue) whereas those involved in photorespiration and calvin cycle were more abundant in young green berries. The levels of proteins in all of these three subgroups declined after veraison. The decrease in abundance of photosynthesis related proteins throughout grape berry development especially after veraison was consistent with previous proteomic studies (Martinez-Esteso et al., 2011; Fraige et al., 2015) and the physiological situation (Pandey and Farmahan, 1977) of developing grape berries.

Proteins associated with lipid metabolism were observed with high frequency and (3.1%, Figure 3B) showed increasing expression after veraison (Figure 3C). Proteins associated with secondary metabolism showed distinct changing patterns. Those involved in phenylpropanoid synthesis were in high abundance in young berries and then decreased during development (Figure 3C in red) whereas those related with later steps of flavonoid and isoprenoid biosynthesis were strongly accumulated after veraison (Figure 3C in purple). Proteins associated with synthesis of N and S containing metabolites were highly expressed around veraison. The distinct arrangement of protein expression reflected the metabolic adjustment during grape berry development.

### Metabolome and proteome data integration

#### Multivariate statistical analyses reveal the trajectory of grape berry development

**Figure 4:**
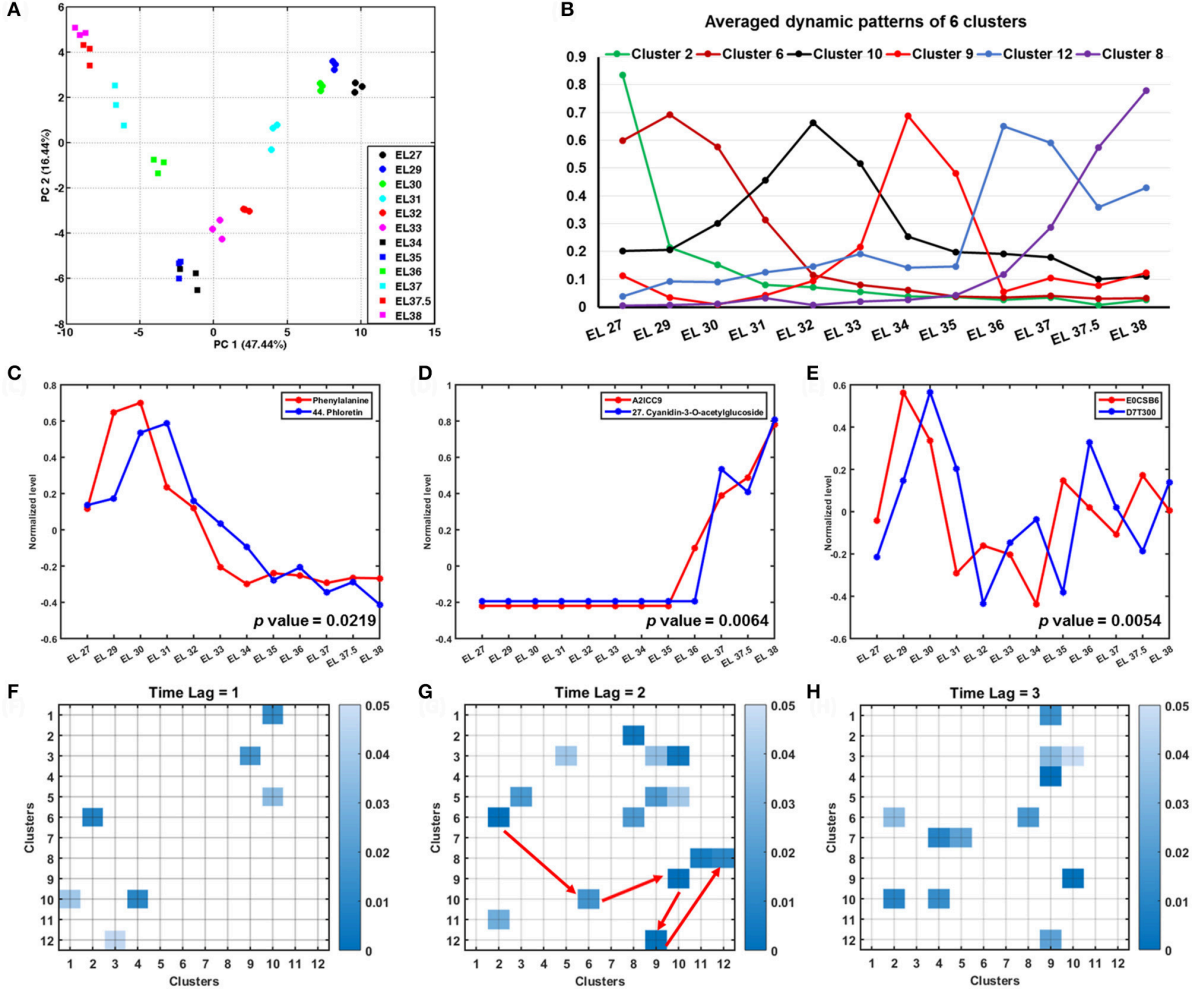
Multivariate statistical and Granger causality analysis of integrated metabolome and proteome data during grape berry development. **(A)** PCA plot of the integrated dataset shows a trajectory during grape berry development. **(B)** The averaged dynamic patterns of 6 clusters from k-means clustering analysis. **(C–E)** Examples of Granger causalities showed directed interactions of metabolite-metabolite, metabolite-protein and protein-protein. **(F–H)** Present Granger causalities between clusters that resulted from k-means clustering analysis with time lag set as 1, 2, and 3, respectively.

The PCA plot (Figure 4A) of the integrated metabolomics and proteomic dataset revealed a continuous trajectory during grape berry development. The separation of various developmental stages indicated a distinction of metabolism on metabolite and protein levels. The first principal component (PC 1) accounting for 47.44% (Figure 4A) of the total variance characterized metabolic and proteomic specificities of grape berries at different developmental stages. Candidates with high absolute loading scores (Table S5) included metabolites, especially flavonoids, caffeic acid, gallic acid, lysine, asparagine, arginine and methionine together with protein candidates involved in development, lipid metabolism, cell wall construction, TCA cycle and protein degradation which accounted for most of the separation among developmental stages.

Further, k-means clustering analysis was applied to group candidates according to their changing patterns. Figure S3 presents the 12 clusters with a bold red line indicating the averaged pattern of all the candidates in each cluster. Cluster 2 and 6 with 86 and 124 candidates, respectively, present candidates with higher abundance at early developmental stages (EL 27 to 31) (Figure 4B, Figure S3). Candidates in these two clusters include amino acids (methionine, phenylalanine, asparagine, ornithine, arginine, lysine, tyrosine), organic acids (caffeic acid, pyruvic acid, gallic acid), sugars and sugar alcohols (arabinose, rhamnose, myo-inositol), (dihydro)flavonol derivatives (epicatechin, 3, 20, 36, 49, 12, 31, 38, 39, 43, 45, compound number see in Table 1) and proteins involved in amino acid metabolism (Table S6). The candidates in cluster 10 and 9 are with highest abundance at EL 32 and veraison (EL 34 and 35), respectively. The most abundant organic acids, i.e., malic acid and citric acid, together with some proteins involved in photosynthesis in cluster 5 were in a relatively higher level during the lag phase and veraison than during the two sigmoidal growth phases. In contrast, those candidates in cluster 3 and 4 showed opposite dynamic patterns including organic acids (shikimic acid, ascorbic acid, fumaric acid, maleic acid), amino acids (valine, leucine, proline isoleucine) and proteins involved in stress response (Figure S3, Table S6). All the annotated anthocyanins and four flavonol derivatives (5, 10, 21, 26, compound number see in Table 1), together with a high abundance of proteins involved in secondary metabolism and lipid metabolism in cluster 1, 8, and 12 mainly accumulated after veraison (EL 36 to 38) (Figure S3, Table S6). The candidates in cluster 7 show a continuous elevation in their abundance whereas those in cluster 11 decreased (Figure S3, Table S6).

#### Granger causation analysis of an integrated metabolomic/proteomic network during grape berry development

Correlation network analysis has been widely applied in omics studies to investigate molecular correlations and connections in metabolism (Steuer et al., 2003; Weckwerth, 2003; Weckwerth et al., 2004a; Sun and Weckwerth, 2012). Those biomolecules with a higher node degree have more connections with other molecules and are therefore regarded as essential connection points in a metabolic network (Weckwerth, 2003; Weckwerth et al., 2004a). The connection degree of nodes might also vary in grape berries at different developmental stages, different cultivars (Cuadros-Inostroza et al., 2016) or under distinct growth conditions (Hochberg et al., 2013; Savoi et al., 2016). Thus, correlation network analysis is an appropriate method for searching important biomolecules involved in specific metabolism processes. Time-lagged correlation analysis by Granger causation represents an advanced level of correlation network analysis (Doerfler et al., 2013). Granger causality analysis was initially introduced by Granger (1969) to predict events based on time series data and time-lagged correlations in economics. It was also applied to some biological studies to interpret directed and nonlinear correlations between metabolites, transcripts and proteins (Walther et al., 2010; Doerfler et al., 2013; Valledor et al., 2014). To further extend the understanding of the dynamic correlations of all the identified metabolites and proteins during the developmental time course, Granger causality analysis was applied to all the candidates and clusters discussed above. The results indicated significant metabolite-metabolite, metabolite-protein and protein-protein correlations. Figure 4C shows the directed correlation of phenylalanine and phloretin (*p*-value = 0.02187) which indicated a strong effect of phenylalanine concentration on phloretin synthesis. Not only precursors but also enzymes showed significant correlation to their product synthesis. One example is the accumulation of anthocyanin synthase (ANS, A2ICC9) prior to anthocyanins (Figure 4D, Table S7). Figure 4E presents a significant granger correlation (*p*-value = 0.00538) between protein E0CSB6 (malate dehydrogenase) and D7T300 (ATPase) indicating the close relationship between TCA cycle and ATP production. Other time series correlations with *p*-values less than 0.05 were summarized in Table S7. The Granger causation network (Figure S4) includes 674 nodes (Table S7) with 21 neighbors in average. Many amino acids (ornithine, arginine, phenylalanine, lysine, tyrosine, asparagine), organic acids (acid_like2, shikimic acid) and their metabolism related proteins (Table S7) show highest node degrees revealing them as potential biochemical hub during grape berry development. The application of Granger analysis to the 12 clusters obtained from the k-means clustering analysis revealed significant time lagged correlations between these clusters. Significant directed connections among these clusters are shown in Figures 4F–H with the time lag set as 1, 2, and 3, respectively (one, two or three time points shifted). The Granger causality correlations from clusters 2 to 6, 6 to 10, 10 to 9, 9 to 12, and 12 to 8 (Figure 4G indicated with red arrows) were visualized as a line chart (Figure 4B) clearly showing their dynamic shift over the developmental time course, especially a significant correlation between cluster 9 and 12. Such combined Granger causality analysis with clustering analysis indicated a general metabolic shift from the metabolism of amino acids, sugars and some flavonoids to organic acid accumulation and finally to lipid and anthocyanin synthesis during grape berry development.

**Figure 5:**
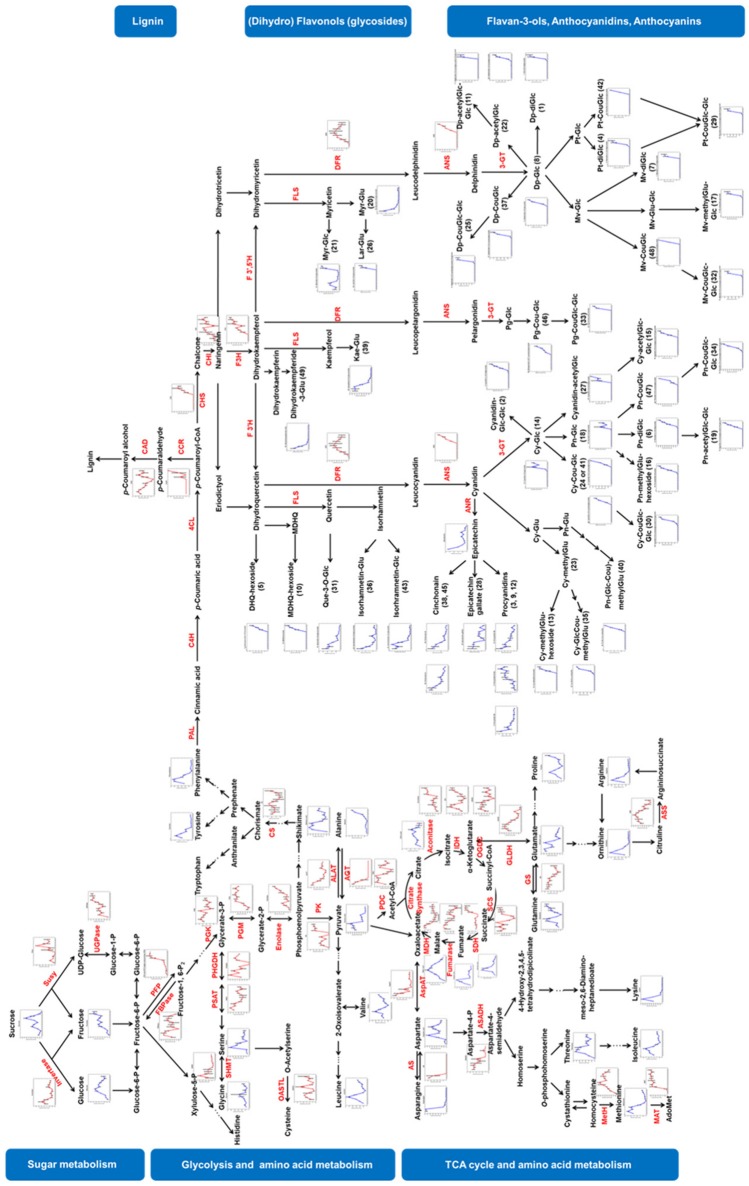
Visualization of metabolite and protein dynamics on their biosynthetic pathways. Metabolites are written in black letters with blue line charts indicating their changing patterns whereas proteins are written in red letters with red line charts. Relative abundance of metabolites and proteins were averaged over three biological replicates. Bars represent standard errors. Susy, sucrose synthase; UGPase, UDP-glucose pyrophosphorylase; PFP, pyrophosphate-fructose 6-phosphate 1-phosphotransferase; FBPase, fructose 1, 6-bisphosphatase; PGK, phosphoglycerate kinase; PGM, phosphoglycerate mutase; PK, pyruvate kinase; PDC, pyruvate dehydrogenase complex; IDH, isocitrate dehydrogenase; OGDC, oxoglutarate dehydrogenase complex; SCS, succinyl coenzyme A synthetase; SDH, succinate dehydrogenase; MDH, malate dehydrogenase; AspAT, aspartate aminotransferase; AS, asparagine synthetase; ASADH, aspartate-semialdehyde dehydrogenase; MetH, methionine synthase; MAT, methionine adenosyltransferase; PHGDH, phosphoglycerate dehydrogenase; PSAT, Phosphoserine transaminase; SHMT, serine hydroxymethyltransferase; OASTL, O-acetylserine (thiol)-lyase; GLDH, Glutamate dehydrogenase; GS, Glutamine synthetase; ASS, argininosuccinate synthase; ALAT, alanine aminotransferase; AGT, alanine-glyoxylate transaminase; CS, chorismate synthase; PAL, phenylalanine ammonia lyase; C4H, cinnamate 4-hydroxylase; 4-coumarate-CoA ligase; CCR, cinnamoyl-CoA reductase; CAD, cinnamyl-alcohol dehydrogenase; CHS, chalcone synthase; CHI, chalcone isomerase; F3H, flavanone 3-hydroxylase; F3′H, flavonoid 3′-hydroxylase; F3′5′H, flavanoid 3′,5′-hydroxylase; FLS, flavonol synthase; DFR, dihydroflavanol 4-reductase; ANS, anthocyanidin synthase; ANR, anthocyanidin reductase; 3-GT, anthocyanidin 3-O-glucosyltransferase.

PCA and k-means clustering analysis presented the systemic dynamics of the metabolites and proteins during grape berry development. To understand the metabolism progress of developing grape berry in a biochemical context, we mapped the sugars, amino acids, organic acids, flavonoids and the related proteins on their corresponding biosynthetic pathways (Figure 5). The integration of dynamics of metabolites as well as proteins involved in both primary and secondary metabolisms presents metabolic checkpoints during grape berry development. This is further discussed below.

## Discussion

### Primary metabolism dynamics

Primary metabolism plays an essential role in grape berry development. The products from primary metabolism pathways are not only crucial for grape survival but also endow grape berry specific characters which are further decisive of its market value.

#### Sugar metabolism

Sugars, especially fructose, glucose and sucrose determine the sweetness of grapes, moreover, the alcohol concentration of wine. In grape berries, sucrose is mainly imported via phloem from source organs. Subsequently, the imported sucrose is either hydrolyzed to glucose and fructose by invertase or converted to glycolysis substrates via sucrose synthase (Susy) and UDP-glucose pyrophosphorylase (UGPase). The fluctuation of sucrose content during grape berry development might be caused by a disproportionate ratio of the import to the consumption. Furthermore, synthesis of sucrose from malate via the gluconeogenic pathway (Ruffner et al., 1975; Dai et al., 2013) might also contribute to the fluctuation of sucrose concentration, especially after veraison. The accumulation of glucose and fructose during grape berry development was reported previously (Wu et al., 2011; Dai et al., 2013). In the present study, fructose accumulated throughout the developmental process whereas glucose concentration did not continue to rise after veraison. Similar glucose dynamics were also observed in some table grape varieties i.e., “Thompson Seedless,” “Crimson Seedless,” and “Red Globe” (Muñoz-Robredo et al., 2011). In contrast to these findings, both glucose and fructose concentration constantly increased during grape berry development in some grape varieties and cultivars (Wu et al., 2011; Dai et al., 2013). The discrepancy in glucose accumulation patterns could be explained by the differences in the ripening process among varieties. The distinct expression pattern of invertase and Susy might explain the unequal accumulation of glucose and fructose. The decline in abundance of invertase since EL 31 caused a decrease in the production of glucose and fructose whereas the increasing expression of Susy ensured the continuous accumulation of fructose.

#### Glycolysis

The substances generated from sugar metabolism are subsequently incorporated into glycolysis. Metabolism along this process generates energy (ATP), reducing equivalents (NADH) as well as intermediates for amino acid biosynthesis, lipids and secondary metabolite production. The abundance of most glycolytic enzymes increased through the development and ripening process (Figure 5). Phosphoglycerate kinase (PGK) was the only glycolytic enzyme, which declined in abundance (Figure 5). The concentration of pyrophosphate-fructose 6-phosphate 1-phosphotransferase (PFP), enolase and pyruvate kinase (PK) in the cytosol strongly increased after veraison (Figure 5). Phosphoglycerate mutase (PGM) was increased during the first sigmoidal growing phase (EL 27 to EL 32) and maintained a relatively constant level afterward. The increase in abundance of glycolytic proteins was consistent with some former reports that studied the proteomic profile of grape skins and berry tissue without seeds (Negri et al., 2008; Kambiranda et al., 2014). However, some studies reported a decrease in abundance of glycolytic enzymes (Davies and Robinson, 2000; Giribaldi et al., 2007; Martinez-Esteso et al., 2011) or glycolytic intermediates (Dai et al., 2013) during berry ripening. Variety and differences in growth conditions might explain these different observations. Additionally, isoforms of enzymes may play different roles at a particular developmental stage (Chaturvedi et al., 2013; Ischebeck et al., 2014; Wang et al., 2016a,b), thus displaying varying dynamics during development. For instance, Fraige et al reported three isoforms of UDPase, of which two candidates decreased in abundance after veraison whereas one increased (Fraige et al., 2015).

#### TCA cycle

The tricarboxylic acid cycle (TCA cycle) generates energy, reducing power and carbon skeletons, which makes it a central hub in metabolism. The Pyruvate dehydrogenase complex (PDC) converts pyruvate to acetyl-CoA which serves as fuel to the TCA cycle. During the grape berry development, PDC was in high abundance during the first growing phase (EL 27-32), followed by a significant decline at the lag phase (EL 32-34) and with a subsequent increase (Figure 5). Similar to PDC, isocitrate dehydrogenase (IDH), oxoglutarate dehydrogenase complex (OGDC), and succinyl coenzyme A synthetase (SCS) were in high abundance during the sigmoidal growth phases whereas in low abundance during the lag phase (Figure 5). The high abundance of these enzymes in the young and ripening berries was consistent with the great demand for energy and building blocks at these two phases. Aconitase and succinate dehydrogenase (SDH) were strongly expressed after veraison. In a former report, a sharp expression of aconitase was observed in ripening grape skin (Negri et al., 2008). Fumarase is the only enzyme whose expression gradually declined throughout the berry development (Figure 5). Malate dehydrogenase (MDH), catalyzing a reversible reaction between oxaloacetate and malate, was concentrated at a lower level at phase I whereas it showed a progressive increase in expression during ripening which was in agreement with previous reports (Martinez-Esteso et al., 2011; Kambiranda et al., 2014). The transcript levels of MDH and malic enzyme were reported to increase during grape berry ripening which might contribute to the decline of malate concentration after veraison (Deluc et al., 2007).

The contents of the intermediates, citrate, succinate, fumarate and malate gradually increased in early stages of development, up to a peak in concentration at EL 31 (fumarate and succinate), EL 33 (malate) or around veraison (citrate) with a subsequent decline. Similar dynamic patterns were observed in developing Cabernet Sauvignon berries with a peak in accumulation of most TCA cycle intermediates at veraison (Dai et al., 2013). The accumulation of these organic acids before veraison was parallel to the high abundance of enzymes at phase I. However, the gradually increasing expression of TCA cycle associated enzymes was accompanied by a decrease in the intermediates during grape berry ripening (phase III). The discrepancy between the increase in the abundance of the enzymes and the decrease in the content of the intermediates during grape berry ripening indicates a high metabolic flux through this pathway with an efficient incorporation of the intermediates in the synthesis of amino acids, lipids and secondary metabolites. In grape, organic acids are responsible for the titratable acidity which is an index for fruit quality. High amounts of organic acids endow young berries a sour taste for defense against herbivores. The organic acids in mature berries are essential for wine production as they protect the fermentation process from bacterial contamination. In wine they are responsible for the sour part of the taste. They are also essential for the color of wine by contributing to the stabilization of anthocyanins (Clemente and Galli, 2011). The changing patterns of those dominant organic acids i.e., malic acid, tartaric acid and citric acid, were consistent with previous reports (Deluc et al., 2007; Ali K. et al., 2011; Muñoz-Robredo et al., 2011; Fraige et al., 2015).

#### Amino acids metabolism

Amino acids are major transportable nitrogenous compounds in grape. In source organs, intermediates from glycolysis and TCA cycle can be utilized as precursors for the synthesis of amino acids. For instance, phosphoenolpyruvate is the precursor of aromatic amino acids that derive from the shikimate pathway; α-ketoglutarate and oxaloacetate are the precursors of glutamate and aspartate family amino acids, respectively. Asparagine and glutamine were the major amino acids in young and mature grape berries respectively. Both of them carry an extra amide group making them efficient nitrogen-carriers. They play important roles in the nitrogen assimilation, transportation and storage in plants. Asparagine and glutamine can be converted to Asp and Glu to serve as precursors for biosynthesis of many other amino acids, e.g., proline, arginine (from glutamate); methionine, threonine and lysine (from aspartate). Several enzymes involved in amino acid metabolism were detected and mapped on Figure 5. Aspartate aminotransferase (AspAT) catalyzes the reversible transfer of an amino group between aspartate and glutamate thus plays an important role in nitrogen distribution. In concordance with a previous report (Martinez-Esteso et al., 2011), AspAT abundancy gradually decreased during grape berry development. Methionine synthase (MetH), catalyzing the synthesis of methionine, is another essential amino acid of the aspartate family. The abundance of MetH increased until veraison, then stayed relatively constant during ripening in the present study. However, it decreased during green developmental stages and increased during ripening in the study of Martinez-Esteso et al. (2011). Glutamine synthetase (GS) is another crucial enzyme involved in nitrogen assimilation. GS, which catalyzes the condensation of glutamate and ammonia to generate glutamine, gradually declined in abundance from EL 27 to EL 36 and slightly increased afterwards. The transcript level of GS was shown to be significantly higher in phase I in a previous study (Deluc et al., 2007). Marinez-Esteso et al reported a decline in the level of GS before veraison (Martinez-Esteso et al., 2011) which is consistent with our result. However, the changing trend of GS after veraison was absent in their study.

### Secondary metabolism dynamics

Grape is rich in polyphenolic compounds that derive from the phenylpropanoid pathway. These bioactive secondary metabolites play essential roles in protecting grape berry against abiotic stresses such as UV radiation (Pontin et al., 2010), high light and high temperature stresses (Ayenew et al., 2015), drought stress (Król et al., 2014) as well as biotic stresses (Gutha et al., 2010; Wallis and Chen, 2012). In addition, they also contribute to organoleptic features of grape berry and wine (Schmidtke et al., 2010; Gutiérrez-Capitán et al., 2014).

#### Lignin

Simple phenolic compounds that are synthesized from phenylalanine can be polymerized to lignin which is an essential component of the cell wall. In the present study, three enzymes, i.e., cinnamoyl-CoA reductase (CCR), cinnamyl-alcohol dehydrogenase (CAD) and ferulate 5-hydroxylase (F5H, Q9M4H8) involved in lignin synthesis were annotated. CCR and CAD, which catalyze the last two steps of monolignol synthesis not only impact lignification but also plant development. Absence of CCR and CAD resulted in dwarfism and sterility in *Arabidopsis* (Thevenin et al., 2011). One protein candidate was annotated as CCR (A5AXM6) and detected after veraison. Four candidates were annotated as CAD. The averaged expression pattern of CAD showed high levels in both young and ripening berries and low level around veraison (Figure 5). Aharoni et al. (2002) reported a comparable pattern of the transcription level of CAD in developing strawberries which are also non-climacteric fruits and undergo color turning phases. In their study, CAD expression level was high in green strawberries followed by a decreasing during the white and turning stages and finally increased again in red strawberries (Aharoni et al., 2002).

#### Flavonoids

Flavonoids are another class of secondary metabolites derived from the phenylpropanoid pathway and share common precursors with lignin. The detected flavonols and flavan-3-ols showed distinct changing patterns with anthocyanins. Parallel phenomena were observed before in other varieties, i.e., Cabernet Sauvigon (Ali M. B. et al., 2011; Degu et al., 2014), Shiraz (Degu et al., 2014) and Norton (*V. aestivalis*) (Ali M. B. et al., 2011) and supposed to be caused by the competition of precursors between anthocyanins and other subfamily members of flavonoids (Ali K. et al., 2011; Degu et al., 2014). The anthocyanin profile obtained from the present data was consistent with that of “Concord” (Liang et al., 2011) and “Pink Sultana” (Boss et al., 1996) in which Cy and Dp derivatives were the dominant anthocyanins. In contrast, Mv derivatives were the most abundant anthocyanins in the other varieties in these two studies (Boss et al., 1996; Liang et al., 2011) and other reports (an overview of all varieties is provided in Table S8) (Mazza et al., 1999; He et al., 2010; Papini et al., 2010; Ali M. B. et al., 2011; Degu et al., 2014, 2015). Noticeably, Early Campbell is a hybrid of *Vitis vinifera* and *Vitis labrusca* by crossing Moore Early with (Belvidere × Muscat of Hamburg). Both Moore Early and Belvidere are seedlings of Concord. The fruit taste and disease resistance of Early Campbell are similar to Concord (Robinson et al., 2012). The similar anthocyanin profile of Early Campbell with that of Concord is probably also due to its genetic background. The annotated proteins that were involved in the flavonoid pathway include chalcone synthase (CHS), chalcone isomerase (CHI), flavanone 3-hydroxylase (F3H), dihydroflavanol 4-reductase (DFR) and anthocyanidin synthase (ANS). CHS catalyzes the condensation of *p*-coumaroyl-CoA with malonyl-CoA to generate chalcone which is further isomerized to naringenin by CHI. Two proteins, annotated as CHS (A2ICC5, G4XGW2), were detected after veraison and increased in abundance during ripening (Table S4). In a previous study, three copies of *Chss* were found in the grape genome. The mRNA levels of *Chs2* and *Chs3* significantly coincided with anthocyanin and *Chs1* and *Chs2* with flavonol biosynthesis (Jeong et al., 2008). The expression of A2ICC5 and G4XGW2 were in accordance with anthocyanin accumulation indicating the involvement of these two CHSs in the coordination of anthocyanin synthesis. The expression level of CHI (A5ANT9) gradually increased before veraison then underwent a sharp decline at veraison (EL 35) with a subsequent recovery to the level before veraison. F3H catalyzes the hydroxylation of flavanones at 3-position to form dihydroflavonols. Both protein candidates annotated as F3H (A2ICC8, A2ICC8) were detected from EL 32 and showed increasing levels during grape berry development. The transcription of *F3hs* appeared to be induced for the biosynthesis of flavonols and anthocyanins (Jeong et al., 2008). Dihydroflavonols are further converted to either flavonols via flavonol synthase (FLS) or to leucoanthocyanidins via DFR. The competition between FLS and DFR influences the contents of flavonols and anthocyanins (Tian et al., 2015) which further affects the color (Lou et al., 2014) and the abilities of plants to cope with stress (Hua et al., 2013; Wang et al., 2013). DFR is a crucial enzyme in the flavonoid pathway involved in the synthesis of anthocyanins, proanthocyanins and tannins (Moyano et al., 1998; Zhang et al., 2008; Hua et al., 2013; Wang et al., 2016c). In the present study, two protein candidates (A5BGJ0, A5BIY8) were annotated as DFR. Their combined expression pattern indicated a progressive increase in abundance of DFR before veraison and a slight decrease afterwards (Figure 5). ANS catalyzing the conversion of colorless leucoanthocyanins to colored anthocyanidins was detected in ripening berries. The abundance of ANS (A2ICC9) significantly increased after veraison (Figure 5) in accordance with the anthocyanin accumulation. MYB-related transcription factors (TFs) are involved in regulation of flavonoid synthesis (Czemmel et al., 2009). It was further reported that VvMYB5b was highly expressed after veraison and the anthocyanin synthesis was enhanced in transgenic tobacco due to ectopic expression of VvMYB5b (Deluc et al., 2008). In our study, four proteins i.e., A5ADL7, A5AHA8, F6GTT4, F6I581 were annotated as “MYB-related transcription factor”. Their summarized content was lowest around veraison whereas the highest level was observed before veraison and during ripening (Table S4 sheet 2). This pattern was neither directly correlated to the amount of anthocyanins in the developing grape berries nor to the protein levels of ANS (A2ICC9). Further we detected 7 bZIP family members (A5B427, A5BZF5, D7SUP9, D7TNE5, F6GTA6, F6GUN1, F6HBQ3). bZIP family member are thought to be involved in the regulation of flavonoid biosynthesis (Malacarne et al., 2016). In a recent study Loyola et al. propose that HY5 and HYH are involved in UV-B-dependent flavonol accumulation in grapevine (Loyola et al., 2016). The concentrations of the bZIP proteins in our study showed an increase toward veraison and a decrease afterwards. Because there is often not a direct dependency between transcriptional and translational/posttranslational control (Nukarinen et al., 2016) it is difficult to compare gene expression levels from other studies with protein levels from our study. Furthermore, the analysis of TFs requires in most cases a specific enrichment step before proteomic analysis. Future investigations will focus more on the discussed transcription factors and their control on developmental processes and flavonoid biosynthesis.

#### Stilbenes

Stilbenes are also derived from the phenylpropanoid pathway and were reported to be enriched in grape. However, we did not detect any stilbene or related enzymes in this study. This might be due to different growth and stress conditions or a different genetic background of the variety we studied. Two publications reported stilbene content in the peel of Early Campbell at veraison stage (Islam et al., 2014; Ahn et al., 2015). They also found that hairy vetch and ryegrass extracts and red and blue LED light induced stilbene accumulation as well as the expression of genes involved in stilbene synthesis. We used the whole berry as the study object which is different from using isolated peel. Furthermore there is evidence that support a competition between the synthesis of stilbenes and flavonoids. One evidence is the negative correlation between resveratrol and anthocyanin accumulation in 5 Vitis species at different developmental species (Jeandet et al., 1995). The other evidence is observed in transgenic strawberries. Hanhineva et al transformed a stilbene synthase gene to strawberries (35S:NS-Vitis3 line). While the STS gene was highly expressed in the transgenic strawberry line, CHS expression was down regulated (Hanhineva et al., 2009). These are two examples indicating the competition relationship between flavonoid and stilbene synthesis pathway. It is thus of interest to compare the flavonoid and stilbene content of this variety under different growth conditions and with other grape varieties that produce stilbenes to investigate the competition of stilbene and flavonoid biosynthesis.

## Conclusion

In summary, the analysis of grape berry development from fruit set to mature fruit by mass spectrometry based platforms revealed intimate correlations between the metabolome and the proteome at the interface of primary and secondary metabolism. The broad coverage of developmental stages included in the present study enabled a dense correlation network analysis of these dynamic processes covering central carbon metabolism such as sugar metabolism, glycolysis, TCA cycle, amino acid metabolism as well as secondary metabolism, especially the flavonoid pathway. Multivariate statistical analysis such as PCA, clustering analysis and Granger causality analysis provides a convenient data mining approach for the interpretation of the integrated metabolome and proteome dataset and revealed the systemic associations between metabolites and proteins during grape berry development. The application of Granger causality analysis is helpful in revealing time-lagged correlations between metabolites and proteins which is especially important for understanding the molecular time-shifts during developmental processes of grape berries. Together with other studies this work provides a reference point for future investigations of grape berry development in a variety of different genotypes.

## Author contributions

LW and WW conceived and designed the experiments. LW performed the experiments. LW, XS, JW, and WW analyzed the data. WW provided the reagents, materials and analytical tools. LW wrote the manuscript. WW and JW revised the manuscript. All the authors approved the final manuscript.

### Conflict of interest statement

The authors declare that the research was conducted in the absence of any commercial or financial relationships that could be construed as a potential conflict of interest.

## Supplementary material

Table S1 Metabolite information.

Table S2 Absolute quantification of 29 metabolite candidates.

Table S3 Protein sequencing information.

Table S4 NSAFs, ANOVA, BLAST results of protein candidates.

Table S5 Loading scores.

Table S6 K-means clustering analysis (12 clusters).

Table S7 Granger causality analysis of the integrated metabolomic and proteomic data sets.

Table S8 Anthocyanin profiles of grape berries of different cultivars.

Figure S1 Relative abundance of anthocyanins in mature berries (EL 38).

Figure S2 Hierachical bi-clustering analysis of protein candidates.

Figure S3 K-means clustering analysis of the integrated dataset.

Figure S4 Granger causality based network.
