# Supplementary figures and images for: System-Level and Granger Network Analysis of Integrated Proteomic and Metabolomic Dynamics Identifies Key Points of Grape Berry Development at the Interface of Primary and Secondary Metabolism
Source: Front Plant Sci. 2017 Jun 30;8:1066. doi: 10.3389/fpls.2017.01066 (PMC5491621; doi:10.3389/fpls.2017.01066)

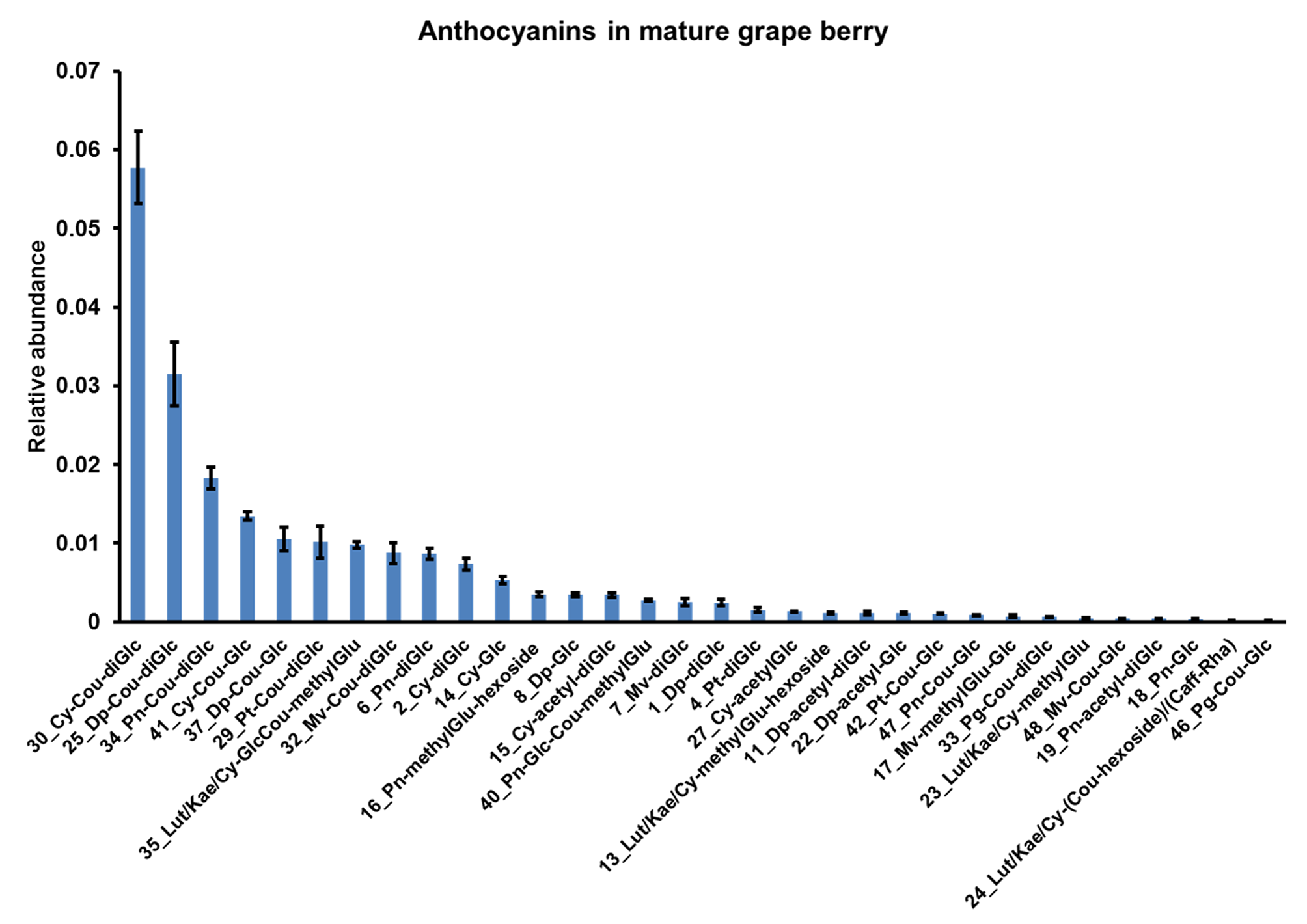

Supplement: Figure S1 — Relative abundance of anthocyanins in mature berries (EL 38). [file Image1.TIF]

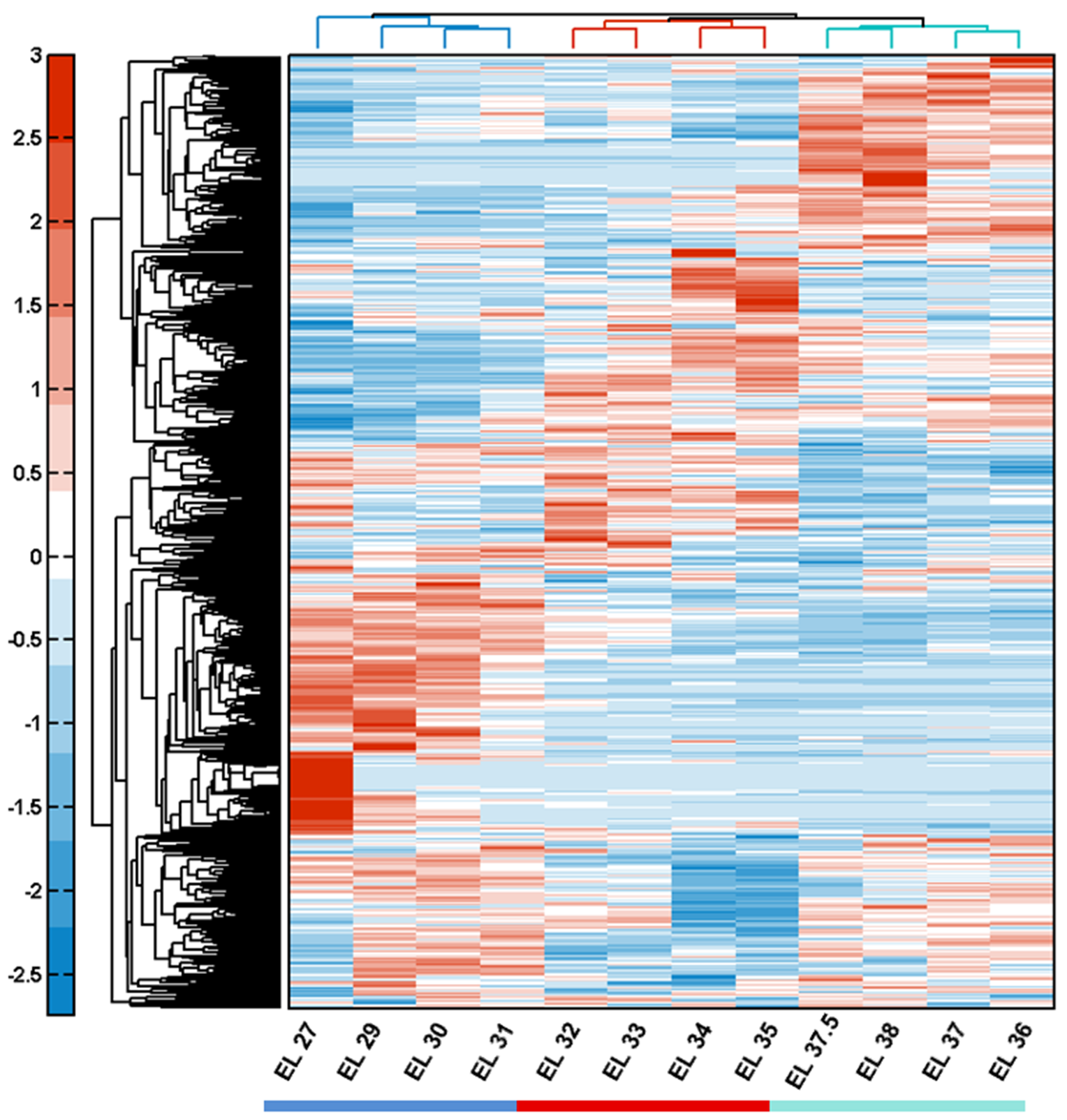

Supplement: Figure S2 — Hierachical bi-clustering analysis of protein candidates. [file Image2.TIF]

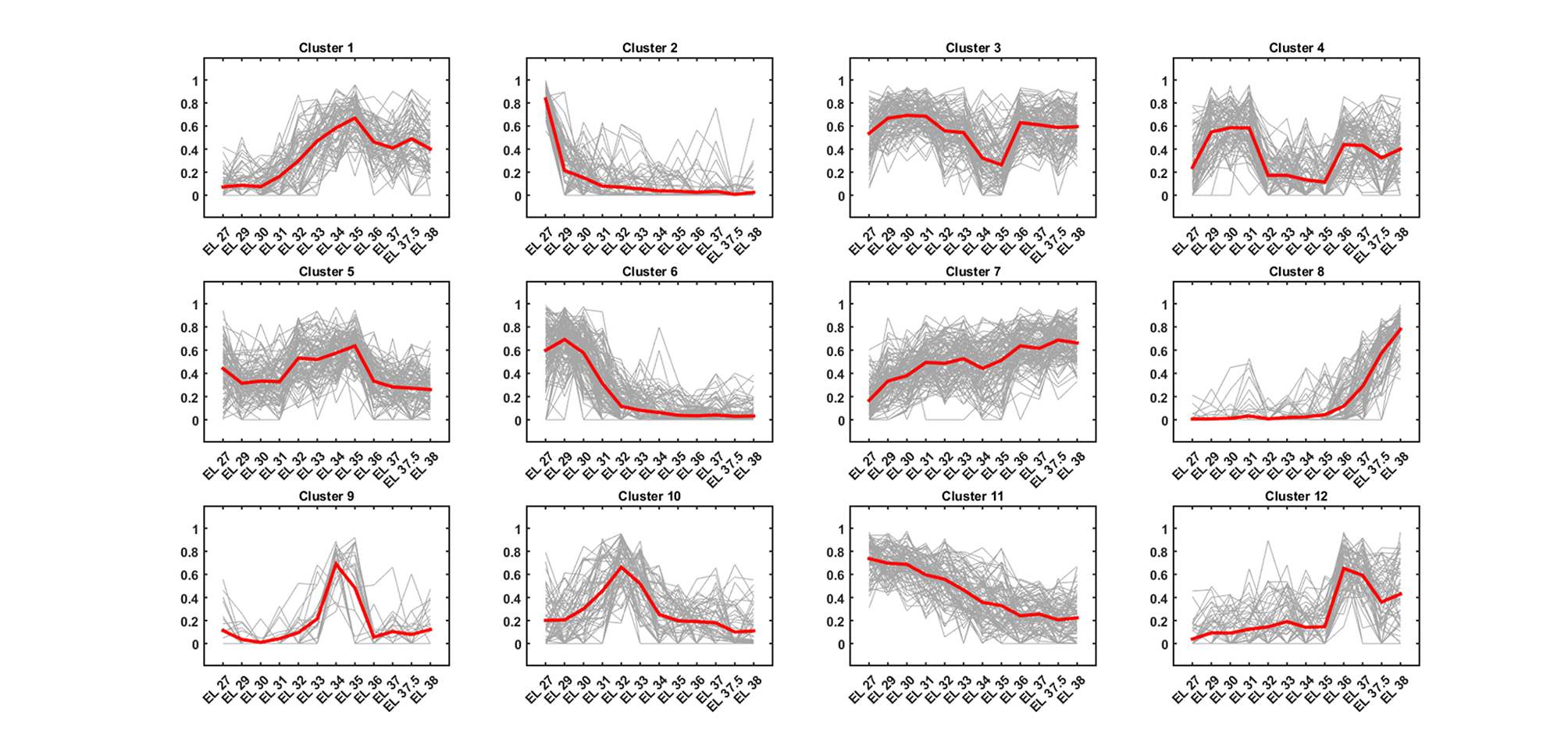

Supplement: Figure S3 — K-means clustering analysis of the integrated dataset. [file Image3.TIF]

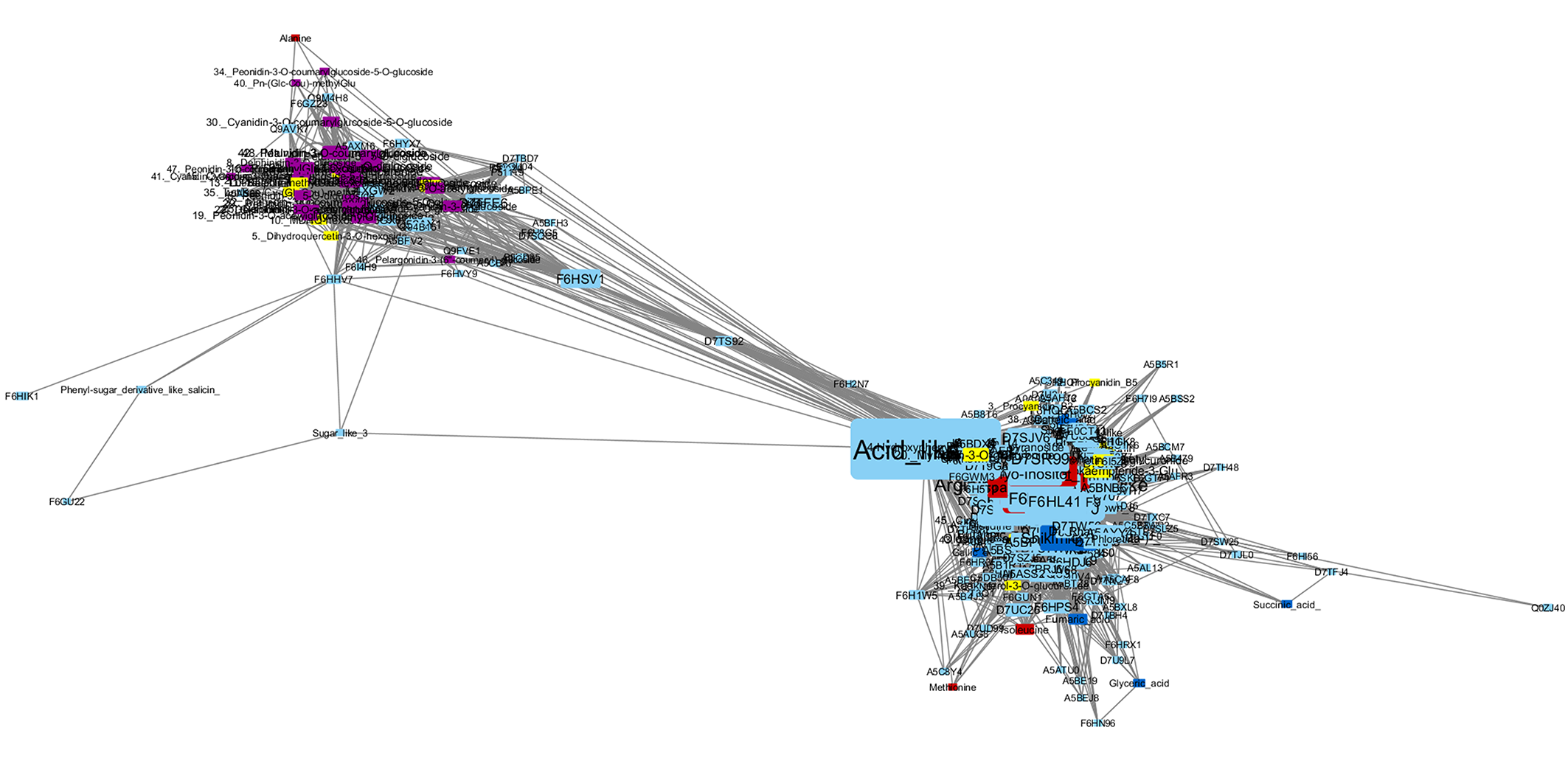

Supplement: Figure S4 — Granger causality based network. [file Image4.TIF]
